# PKMYT1 as a Potential Target to Improve the Radiosensitivity of Lung Adenocarcinoma

**DOI:** 10.3389/fgene.2020.00376

**Published:** 2020-04-29

**Authors:** Huan-ping Long, Jia-qing Liu, Yang-yang Yu, Qiao Qiao, Guang Li

**Affiliations:** Department of Radiation Oncology, The First Affiliated Hospital of China Medical University, Shenyang, China

**Keywords:** bioinformatics, PKMYT1, G2 checkpoint, lung adenocarcinoma, radioresistance

## Abstract

**Objective:**

This article is dedicated to finding important genes related to the prognosis of lung adenocarcinoma (LUAD), looking for a new gene that may affect tumor radiosensitivity, and conducting basic experiments to verify the relationship between this gene and the radiosensitivity of LUAD.

**Methods:**

The gene expression profiles GSE32863, GSE33532, and GSE43458 were obtained from NCBI-GEO. GEO2R and a Venn diagram were used to identify upregulated genes. STRING and Cytoscape were applied to develop a protein–protein interaction network (PPI) and analyze the modules. The Database for Annotation, Visualization and Integrated Discovery (DAVID) was used to process the GO and KEGG pathway analysis. The Kaplan Meier plotter and Gene Expression Profiling Interactive Analysis (GEPIA) were applied to get the significant prognostic information and differential expression between LUAD tissues and normal lung tissues. Western blotting and Q-PCR were used to detect the expression of PKMYT1 in tissues. Small interfering RNAs (siRNAs) were used to knockdown PKMYT1. The colony survival experiment was used to assess the effect of PMYT1 on the radiosensitivity of tumor cells. Cell cycle analysis was used to assess cell cycle distribution.

**Results:**

We identified 14 genes (PKMYT1, TTK, CHEK1, CDC20, PTTG1, MCM2, CDC25C, MCM4, CCNB1, CDC45, MAD2L1, CCNB2, BUB1, and CCNA2) that are important for LUAD and may be potential therapeutic targets. We confirmed that PKMYT1 is highly expressed in LUAD and firstly demonstrated that artificially silencing the expression of PKMYT1 can abrogate IR-induced G2/M phase arrest and increase the sensitivity of cancer cells to radiation.

**Conclusion:**

In summary, we obtained 14 core genes related to the poor prognosis of LUAD via bioinformatical analysis. We identified that PKMYT1 was significantly upregulated in LUAD tissues and firstly demonstrated that knockdown of PKMYT1 can eliminate the radiation-induced G2/M arrest, resulting in a lower survival rate for cells receiving radiation therapy. Our findings suggested that PKMYT1 is a promising target to improve the radiosensitivity of LUAD.

## Introduction

Lung cancer accounts for 18.4% of cancer deaths and remains the leading cause of cancer-related deaths (Bray et al., 2018). Lung adenocarcinoma has become one of the most common types of lung cancer in recent decades (Siegel et al., 2018). Radiotherapy is an effective treatment of LUAD, especially when some patients are not suitable for surgery. However, LUAD from different patients may display different degrees of radiation tolerance. Radioresistance is the main factor reducing the effectiveness of radiotherapy, resulting treatment failure (Provencio et al., 2010; Le Pechoux, 2011). Thus, it is important to conduct researches on developing radiosensitizers to treat this lethal disease.

Bioinformatics can help us explore a lot of valuable clues, find meaningful genes, and conduct new research. In the present study, GSE32863, GSE33532, and GSE43458 were analyzed through bioinformatics. Finally, we obtained fou14 genes (PKMYT1, TTK, CHEK1, CDC20, PTTG1, MCM2, CDC25C, MCM4, CCNB1, CDC45, MAD2L1, CCNB2, BUB1, and CCNA2), which were significantly enriched in the cell cycle.

After an extensive literature review of these 14 genes, we found that PKMYT1 is a highly promising gene that may be closely related to tumor radiosensitivity. This is because studies have found that the abrogation of G2 checkpoint effectively reduced radiation-induced cell cycle arrest and increased tumor radiosensitivity (Leijen et al., 2010; De Witt et al., 2011; Qin et al., 2014; Busch et al., 2017). Additionally, PKMYT1 is currently considered to be the target of G2 checkpoint elimination and mitotic catastrophe (Tse et al., 2009; Schmidt et al., 2017) because PKMYT1 can prevent cells from transitioning from G2 to mitosis phase in two ways. One is inhibiting Cdk1 activity via phosphorylating Cdk1 at Thr14 and Tyr15, and the other is preventing the Cdk1-CycB complex from entering the nucleus by binding to the Cdk1-CycB complex and sequestering it in the cytoplasm (Fattaey and Booher, 1997; Wells et al., 1999; Passer et al., 2003).

However, there is no experimental evidence to prove the relationship between PKMYT1 and tumor radiosensitivity. We designed experiments to see if PKMYT1 can affect the resistance of LUAD cells to radiation, providing important evidence for PKMYT1 as a radiosensitizer in radiotherapy.

## Materials and Methods

### Bioinformatics Analysis

The GSE32863, GSE33532, and GSE43458 were obtained from NCBI-GEO, which included 40 LUAD tissues and 10 normal lung tissues, 80 LUAD tissues and 30 normal lung tissues, and 58 LUAD tissues and 58 normal lung tissues, respectively. GEO2R was used to identify upregulated genes with logFC > 0.5 and an adjusted *P* < 0.001. Venn diagram was used to select upregulated genes co-expressed in these three gene chips. A PPI network was established by STRING. Then, we use the Cytoscape to find potential relevance between those genes. Next, we used MCODE (Molecular Complex Detection) to obtain core genes. We processed BP, MF, CC, and KEGG pathway analyses (*P* < 0.05) using the Database for Annotation, Visualization and Integrated Discovery (DAVID). The Kaplan Meier plotter and Gene Expression Profiling Interactive Analysis (GEPIA) were applied to get the significant prognostic information (*P* < 0.05) of hub genes in LUAD and their differential expression between LUAD tissues and normal lung tissues (*P* < 0.05).

### Human LUAD Specimens

Human LUAD tissues with the matched normal adjacent specimens were obtained from The First Hospital of China Medical University. This study was approved by the Ethics Committee of The First Hospital of China Medical University.

### Cell Culture

LUAD cell lines: A549, H299, H1975, H1650, and H441 were cultured using Roswell Park Memorial Institute-1640 (RPMI-1640, Hyclone) medium containing 10% fetal bovine serum (FBS, Clarks) at 37°C.

### siRNA and Transfection

Small interfering RNAs (siRNAs) against PKMYT1 (siRNA#1 and siRNA#2) and negative control siRNAs (NC-siRNA), which were obtained from Shanghai GENECHEM Co., Ltd. (Jikai, Shanghai, China), were transfected into A549 and H1299 cells using Neofect reagent (ProbeGenne, Jiangsu, China) according to the manufacturer’s instructions.

### Quantitative PCR (Q-PCR)

Reverse transcription was carried out according to the instructions of PrimeScript^TM^RT reagent Kit with GNA Eraser (Takara), and SYBR Premix Ex TaqII (Takara) was used for Q-PCR. We used several sequences: PKMYT1 forward primer 5′-CATGGCTCCTACGGAGAGGT-3′ and reverse primer 5′-ACATGGAACGCTTTACCGCAT-3′; β-actin forward primer 5′-CCAGACAGCACTGTGTTGGCATA-3′, and reverse primer 5′-ATGTTGCCCTAGACTTCGAGCAAG-3. The results were calculated using the 2^–ΔΔCt^ method.

### Western Blotting Assay

The protein samples were loaded onto SDS-PAGE, transferred onto PVDF membranes, and immunoblotted with primary antibodies against PKMYT1 (Abcam) and β-actin (CST) and peroxidase-labeled secondary antibodies (CST). The Enhanced Chemiluminescence System (OLYMPUS) was used to display immune response bands.

### Irradiation Treatment and Clonogenic Survival Assay

Transfected A549 and H1299 cells were irradiated with 0, 2, 4, 6, and 8 Gy X-rays. After 12 days, the cells were fixed with 4% paraformaldehyde and stained with 0.1% crystal violet solution. A single- hit multi-target model formula: SF = 1–(1-e^–D/D0^)^n^ was used to fit cell survival curves and calculate parameters (D0, Dq, k, N, and SER).

### Cell Cycle Analysis

Cells transfected with siRNA # 1, siRNA # 2, and NC-siRNA were irradiated with X-rays at different doses (0, 4Gy). After irradiation, the cells were further cultured for 24 h. The cells were trypsinized, washed with PBS, fixed with ice-cold 70% ethanol, and stored at 4°C for 12–24 h. Prior to analysis by flow cytometry, the cells were washed with PBS, treated with 0.25 mg/ml RNase A, and 50 μg/ml propidium iodide for 30 min at 37°C. Next, the cells were subjected to cell cycle analysis using a fluorescence-activated cell sorter (BD FACS Calibur).

### Statistical Analysis

Statistical analysis was conducted by SPSS 20.0 software. The statistical significance between groups was determined using a two-tailed Student’s *t*-test. One-way ANOVA was used to compare different time points/dose within the same group. *P* < 0.05 were considered to indicate statistically significant differences.

## Results

### Identification of Upregulated Genes in LUAD

**FIGURE 1:**
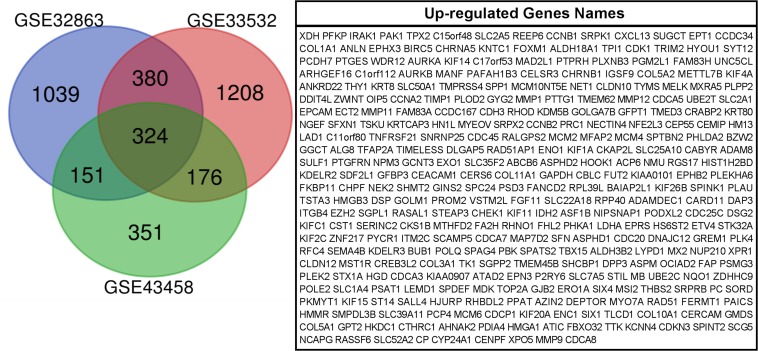
324 commonly up-regulated genes were obtained from GSE33532, GSE43458and GSE 32863 via GEO2R online tools and Venn diagram software.

We used GEO2R to filter 2707, 1082, and 2085 upregulated genes from GSE33532, GSE43458, and GSE32863, respectively. We identified 324 commonly upregulated genes in the above datasets via Venn diagram software (Figure 1).

### Analyses of Gene Ontology and Pathway Enrichment

DAVID was used to analyze the KEGG pathway and GO analysis of 324 genes. The GO analysis showed that, for biological processes (BP), genes significantly enriched DNA unwinding involved in DNA replication, collagen fibril organization, microtubule-based movement, DNA replication initiation, mitotic spindle assembly, and mitotic metaphase plate congression. For molecular function (MF), genes were primarily enriched in ATP binding, microtubule motor activity, and ER retention sequence binding. For cellular component (CC), genes were particularly enriched in the kinesin complex, midbody, and chromosome passenger complex. The results of the KEGG pathway analysis indicated that the 324 genes were significantly enriched in the cell cycle, biosynthesis of amino acids, and fructose and mannose metabolism (Table 1).

**TABLE 1:** Gene ontology analysis of 324 genes.

**Categories**	**Term**	**Count**	**%**	***p*-value**	**FDR**
GOTERM_BP_DIRECT	GO:0006268~DNA unwinding involved in DNA replication	5	0.009586073	9.71E-06	0.015322831
	GO:0030199~collagen fibril organization	7	0.013420503	1.80E-05	0.028344256
	GO:0007018~microtubule-based movement	9	0.017254932	2.75E-05	0.043412072
	GO:0006270~DNA replication initiation	5	0.009586073	6.78E-04	1.065044432
	GO:0090307~mitotic spindle assembly	5	0.009586073	0.001347575	2.105625858
	GO:0007080~mitotic metaphase plate congression	5	0.009586073	0.001568185	2.446354599
GOTERM_CC_DIRECT	GO:0005871~kinesin complex	9	0.017254932	2.72E-06	0.003474135
	GO:0030496~midbody	11	0.021089361	2.88E-06	0.003685049
	GO:0032133~chromosome passenger complex	4	0.007668859	6.78E-05	0.086752849
GOTERM_MF_DIRECT	GO:0005524~ATP binding	44	0.084357446	5.39E-06	0.007214573
	GO:0003777~microtubule motor activity	7	0.013420503	6.96E-04	0.927666683
	GO:0046923~ER retention sequence binding	3	0.005751644	0.00101958	1.355274839
KEGG_PATHWAY	cfa04110:Cell cycle	17	0.032592649	2.86E-09	3.49E-06
	cfa01230:Biosynthesis of amino acids	11	0.021089361	1.68E-06	0.002040968
	cfa00051:Fructose and mannose metabolism	6	0.011503288	3.68E-04	0.446985975

### PPI Network and Module Analysis

**FIGURE 2:**
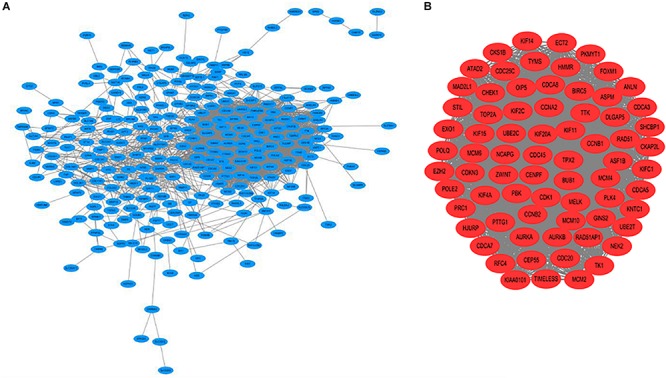
**(A)** Construction of the protein–protein interaction (PPI) network. **(B)** Identification of the significant module.

A PPI network was constructed which included 267 nodes and 3161 edges. Then, a significant module with 72 nodes and 2357 edges was identified via MCODE (Figure 2).

### Identification of Core Genes

**FIGURE 3:**
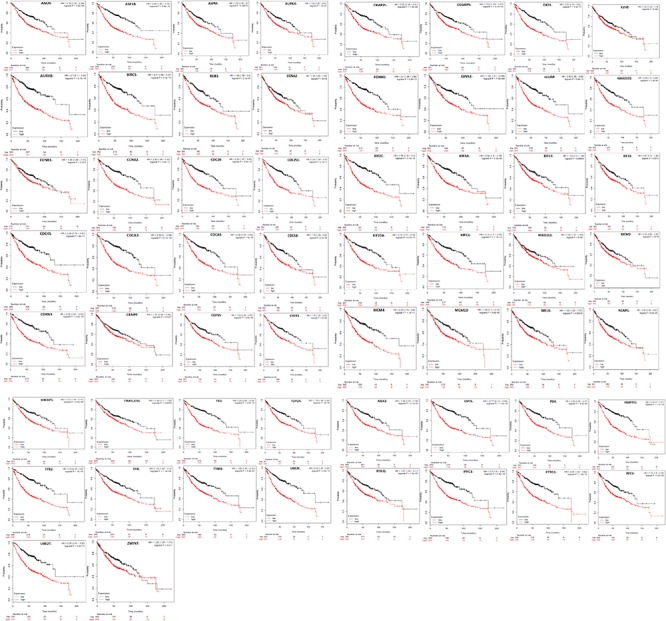
A total of 58 of 72 genes had a significantly poor survival rate in LUAD via Kaplan meier plotter online tools (*P* < 0.05).

**FIGURE 4:**
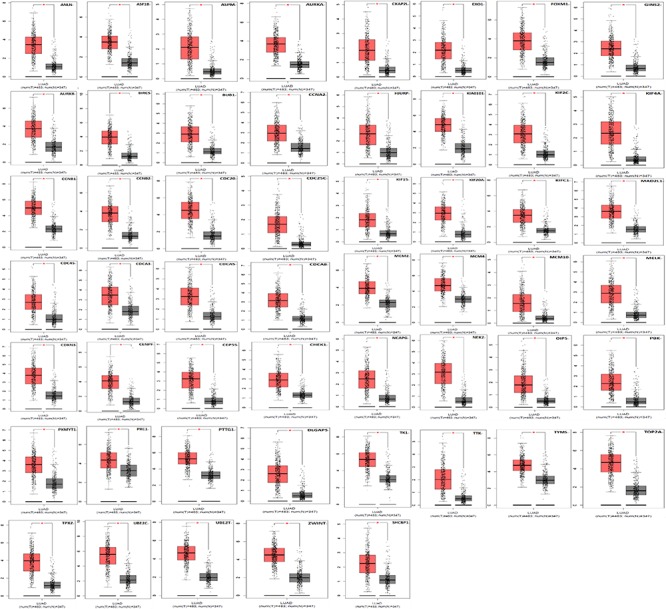
A total of 53 of 58 genes are significantly upregulated in LUAD samples contrasted to normal lung samples (*P < 0.05).

Kaplan Meier plotter was used to analyze the overall survival (OS) of 72 hub genes. We identified that 58 genes had a significantly poor OS in LUAD (Table 2 and Figure 3). Next, we found that 53 of 58 genes are highly expressed in LUAD tissues compared to normal lung tissues (Table 3 and Figure 4) via GEPIA.

**TABLE 2:** The overall survival analysis of the 72 hub genes.

**Categories**	**Genes names**
Genes with significantly poor OS (*P* < 0.05)	MAD2L1 PKMYT1 KIF2C TTK CCNA2 BUB1 KIF20A CKAP2L BIRC5 CCNB2 EXO1 CDCA3 CDKN3 CDC20 OIP5 HJURP KIF14 MCM2 MCM10 PRC1 MELK KIF15 NCAPG PTTG1 TK1 KIF4A UBE2C FOXM1 AURKA KIFC1 CEP55 CDC25C TYMS ASF1B KIAA0101 EZH2 CENPF DLGAP5 CHEK1 CDCA5 ZWINT ANLN POLQ CDC45 GINS2 TIMELESS SHCBP1 RFC4 NEK2 AURKB UBE2T CCNB1 TPX2 MCM4 TOP2A PBK ASPM CDCA8
Genes without significantly poor OS (*P* > 0.05)	KNTC1 HMMR RAD51 POLE2 ECT2 RAD51AP1 STIL ATAD2 PLK4 CDK1 KIF11 MCM6 CDCA7 CKS1B

**TABLE 3:** Differential expression of 53 genes between LUAD and normal lung tissues.

**Categories**	**Genes names**
Genes with significantly high expression in LUAD (*P* < 0.05)	MAD2L1 PKMYT1 KIF2C TTK CCNA2 BUB1 KIF20A CKAP2L BIRC5 CCNB2 EXO1 CDCA3 CDKN3 CDC20 OIP5 HJURP MCM2 MCM10 PRC1 MELK KIF15 NCAPG PTTG1 TK1 KIF4A UBE2C FOXM1 AURKA KIFC1 CEP55 CDC25C TYMS ASF1B KIAA0101 CENPF DLGAP5 CHEK1 CDCA5 ZWINT ANLN CDC45 GINS2 SHCBP1 NEK2 AURKB UBE2T CCNB1 TPX2 MCM4 TOP2A PBK ASPM CDCA8
Genes without significantly high expression in LUAD (*P* < 0.05)	KIF14 EZH2 POLQ TIMELESS RFC4

### KEGG Pathway Analysis

The results of the KEGG pathway analysis indicated that the 53 genes were significantly enriched in cell cycle, oocyte meiosis, and progesterone-mediated oocyte maturation. Results showed that fourteen genes (PKMYT1, TTK, CHEK1, CDC20, PTTG1, MCM2, CDC25C, MCM4, CCNB1, CDC45, MAD2L1, CCNB2, BUB1, and CCNA2) enriched in cell cycle (Table 4).

**TABLE 4:** KEGG pathway enrichment analysis of 53 genes.

**Term**	**Count**	**%**	***p*-value**	**genes**
cfa04110:Cell cycle	14	0.15171218	2.29E-18	PKMYT1, TTK, CHEK1, CDC20, PTTG1, MCM2, CDC25C, MCM4, CCNB1, CDC45, MAD2L1, CCNB2, BUB1, CCNA2
cfa04114:Oocyte meiosis	8	0.086692674	2.13E-08	CCNB2, MAD2L1, BUB1, PKMYT1, CDC20, AURKA, PTTG1, CDC25C
cfa04914: Progesterone-mediated oocyte maturation	7	0.07585609	1.67E-07	CCNB1, CCNB2, MAD2L1, BUB1, PKMYT1, CDC25C, CCNA2

After an extensive literature review of these 14 genes, we found that PKMYT1 is a highly promising gene that may be closely related to tumor radiosensitivity. However, there is no experimental evidence to prove the relationship between PKMYT1 and tumor radiosensitivity. We therefore conducted basic experiments to explore whether PKMYT1 can affect tumor radiosensitivity.

### PKMYT1 Is Highly Expressed in Tumors Contrasted to Normal Tissues Derived From LUAD Patients

**FIGURE 5:**
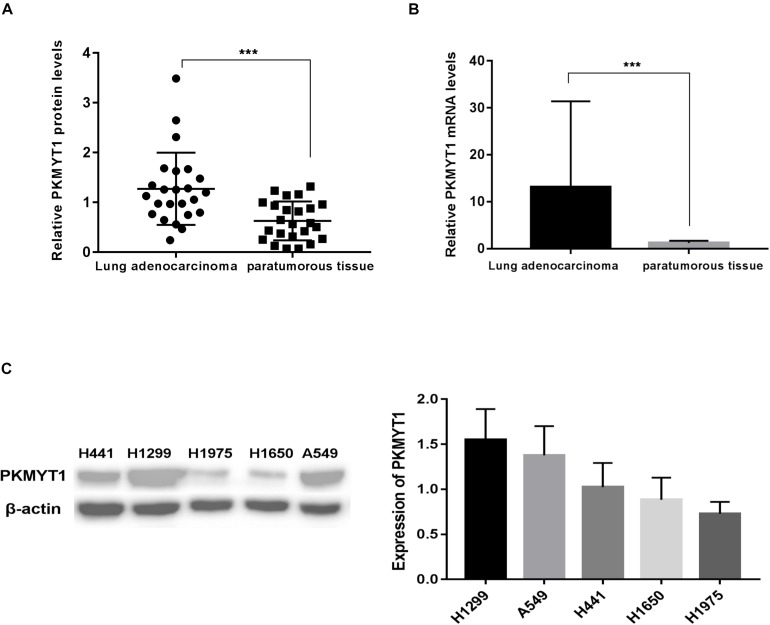
PKMYT1 is highly expressed in lung adenocarcinoma tissues. **(A)** Relative PKMYT1 protein levels in 24 pairs of lung adenocarcinoma tissues and their matched normal tissues were determined by western blotting. **(B)** Relative PKMYT1 mRNA levels in 33 pairs of lung adenocarcinoma tissues and their matched normal tissues were determined by Q-PCR. **(C)** Expression level of PKMYT1 in five lung adenocarcinoma cell lines. ***p < 0.001.

Figure 5A reveals that the protein levels of PKMYT1 were distinctly increased in LUAD tissues compared to matched normal lung tissues via western blotting. Figure 5B shows that the mRNA levels of PKMYT1 in LUAD tissues were significantly higher than those in adjacent normal lung tissues by Q-PCR.

### siRNA Mediated the Silence of PKMYT1

**FIGURE 6:**
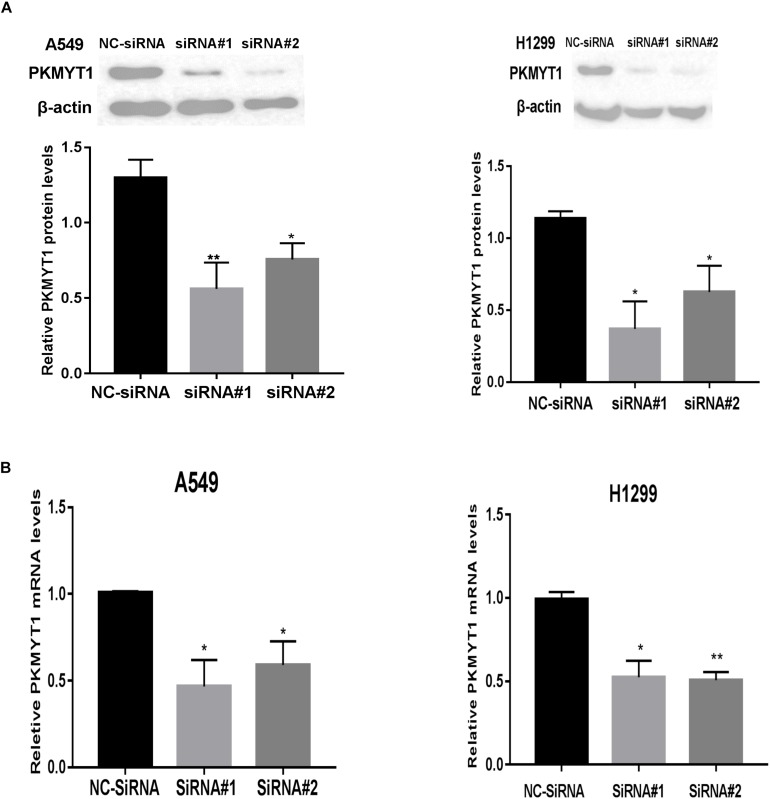
Silencing efficiency of PKMYT1 in A549 and H1299 cells. **(A)** Western blotting assays detected the relative PKMYT1 protein levels in A549 and H1299 cells after transfecting with siRNA#1, siRNA#2, and NC-siRNA. **(B)** Q-PCR assays detected the relative PKMYT1 mRNA levels in A549 and H1299 cells after transfecting with siRNA#1, siRNA#2, and NC-siRNA. *p < 0.05, **p < 0.01.

The expression of PKMYT1 in LUAD cell lines, including A549, H299, H1975, H1650, and H441, was confirmed by WB (Figure 5C). We next use the two most expressive ones (A549 and H1299 cells) for follow-up experiments. Both cells were infected with either control or PKMYT1 siRNA. Then, the Q-PCR and western blotting analyses were conducted, and the data suggested that siRNAs can significantly inhibit the expression of PKMYT1 in RNA and protein levels (Figures 6A,B).

### Downregulation of PKMYT1 Is Significantly Associated With Radiosensitivity of LUAD Cells

**FIGURE 7:**
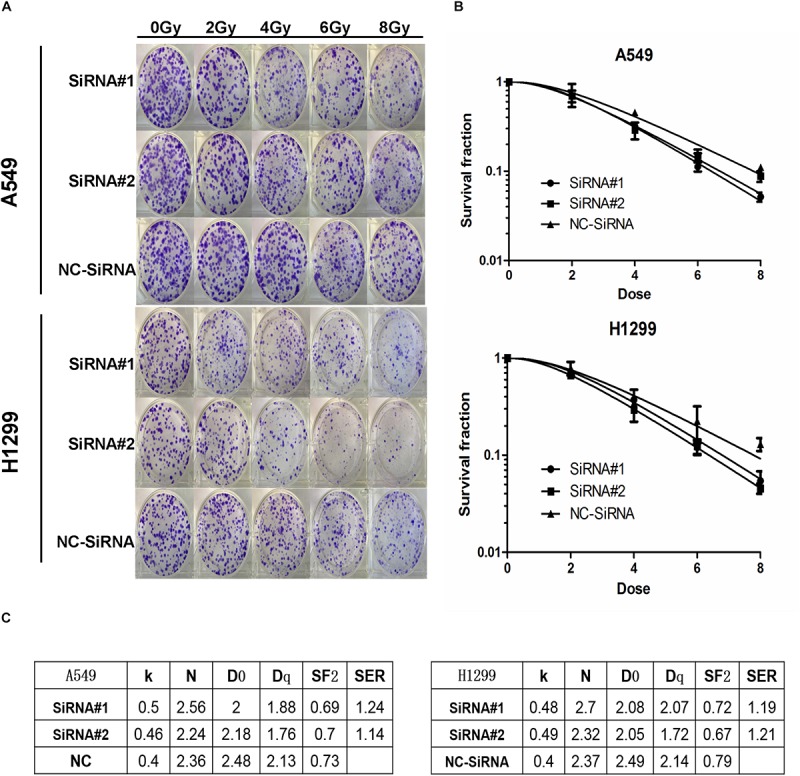
PKMYT1 silencing results in enhanced radiosensitivity of lung adenocarcinoma cells. **(A)** Representative images of colony formation in A549 and H1299 cells pretreated with SiRNA#1, SiRNA#2, or NC-SiRNA for 48 h and then exposed to 2, 4, 6, or 8 Gy of X-ray irradiation. **(B)** Clonogenic cell survival curves were generated for A549 and H1299 cells treated with SiRNA#1, SiRNA#2, or NC-SiRNA after radiation. The survival data were normalized to those of the unirradiated control cells. **(C)** The single-hit multitarget model to calculate the radiobiological parameters of A549 and H1299 cell lines after downregulating PKMYT1 expression.

The radiosensitivity of transfected A549 and H1299 cells was examined by colony formation assay. The data in Figure 7 demonstrates that artificial silencing of PKMYT1 remarkably enhanced radiosensitizing properties in LUAD Cells.

### Downregulation of PKMYT1 Alters Cell Cycle Distribution and Abrogates IR-Induced G2/M Phase Arrest

**FIGURE 8:**
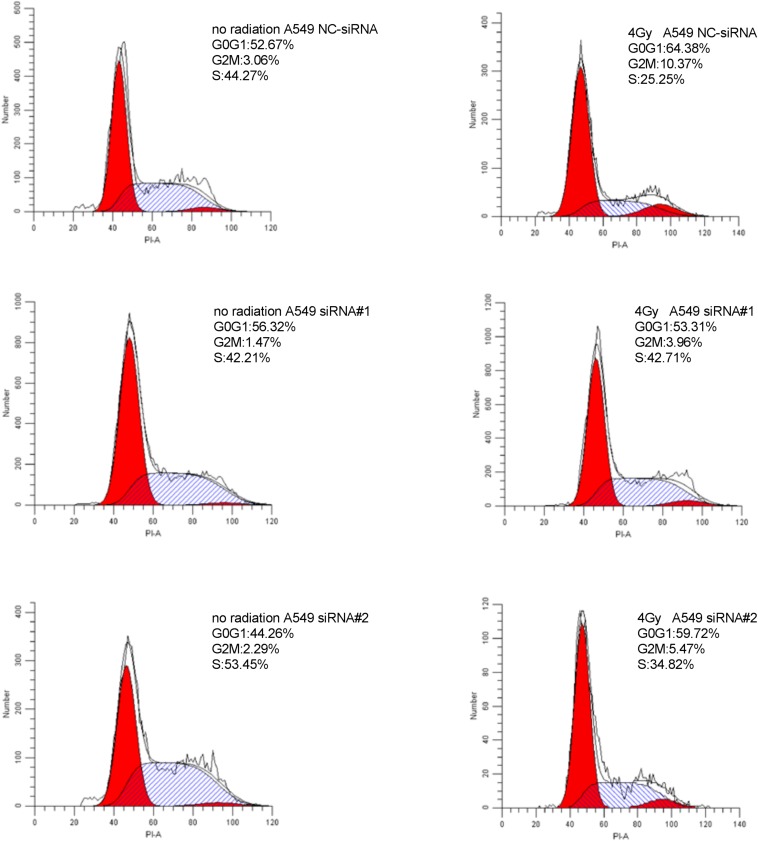
Downregulation of PKMYT1 abolishes irradiation-induced G2/M arrest in A549 cells.

**FIGURE 9:**
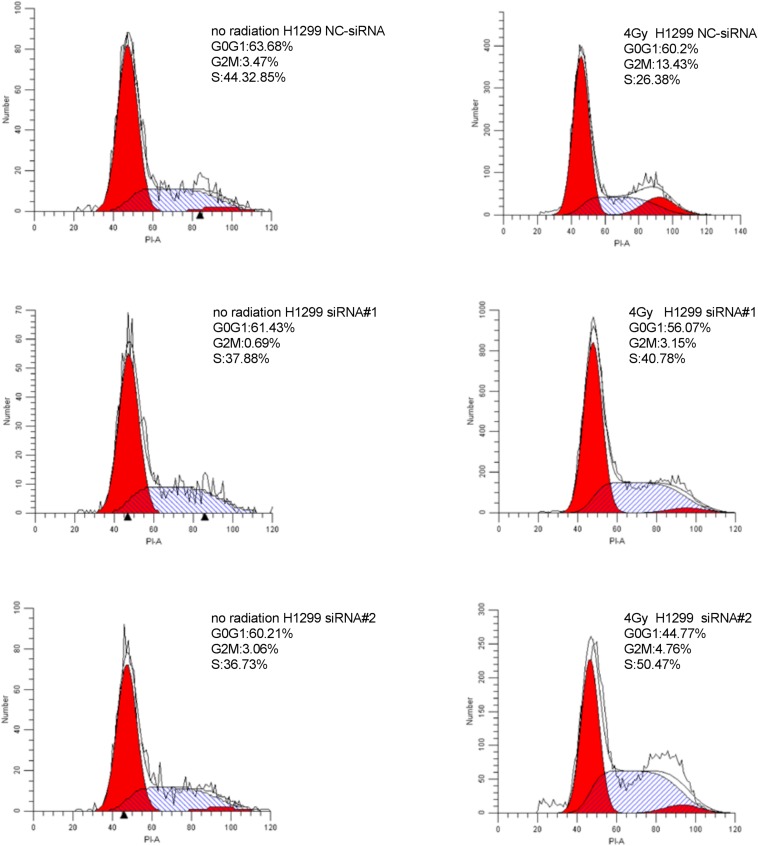
Downregulation of PKMYT1 abolishes irradiation-induced G2/M arrest in H1299 cells.

As shown in Figures 8, 9, silencing of PKMYT1 can decrease the G2/M cell proportion in A549 and H1299, and the difference was statistically significant (*P* < 0.05). In both cell lines, following treatment with silencing of PKMYT1 + radiation (4Gy of X-rays), the cells also accumulated in G2/M, but to a much lower level compared to the radiation control (*p* < 0.05), suggesting that the silencing of PKMYT1 can eliminate the radiation-induced G2/M arrest, thereby improving the radiosensitivity of tumor cells.

## Discussion

The identification of a target to improve the radiosensitivity of LUAD is significant. In this article, we obtained 14 core genes related to the prognosis of LUAD by analyzing GSE32863, GSE33532, and GSE43458. After an extensive literature review of these 14 genes, we found that PKMYT1 is a highly promising gene that may be closely related to tumor radiosensitivity. This is because PKMYT1 is currently considered to be the target of G2 checkpoint elimination and mitotic catastrophe (Tse et al., 2009; Schmidt et al., 2017) as it can prevent cells from transitioning from G2 to mitosis phase by affecting the activity of Cdk1 and the nuclear shuttle of the Cdk1-CycB complex (Fattaey and Booher, 1997; Wells et al., 1999; Passer et al., 2003). Additionally, the abrogation of the G2 checkpoint effectively reduced radiation-induced cell cycle arrest and increased tumor radiosensitivity (Leijen et al., 2010; De Witt et al., 2011; Qin et al., 2014; Busch et al., 2017). However, the functional importance of PKMYT1 in tumor radiosensitivity has not yet been reported.

In this study we demonstrated that PKMYT1 is highly expressed in LUAD tissues contrasted with normal lung tissues. We found that artificially silencing the expression of PKMYT1 can increase the sensitivity of LUAD cells to radiation. Cell cycle analysis found that downregulation of PKMYT1 alters cell cycle distribution and abrogates IR-induced G2/M phase arrest.

Most cancer types fail to establish the G1 checkpoint, and this is usually because of mutation or deletion of the p53 tumor suppressor. Hence, cells deficient in p53 are more vulnerable to inactivation of the G2 checkpoint (Passer et al., 2003). As a consequence, therapeutic ablation of G2 checkpoints may preferentially make p53-deficient tumor cells sensitive to DNA damaging treatments while retaining normal cells with intact p53 function (Dixon and Norbury, 2002; Meng et al., 2018). In addition, a very interesting point is that research has found that PKMYT1 is not necessary for the cell cycle of normal cells, but it has a rate-limiting function for the recovery of checkpoints after DNA damage (van Vugt et al., 2004; Chow and Poon, 2013). Inhibitors of components essential for the progression of cell cycle progression in normal cells may be limited by their greater toxicity to normal cells when subjected to anti-tumor therapy. Since PKMYT1 is relatively less important for normal cell cycle progression, PKMYT1 is a particularly attractive target for anticancer therapy.

## Conclusion

In summary, we obtained 14 core genes (PKMYT1, TTK, CHEK1, CDC20, PTTG1, MCM2, CDC25C, MCM4, CCNB1, CDC45, MAD2L1, CCNB2, BUB1, and CCNA2) related to the poor prognosis of LUAD via bioinformatical analysis. We identified that PKMYT1 was significantly upregulated in LUAD tissues and firstly demonstrated that knockdown of PKMYT1 resulted lower survival rate when cells receiving radiation therapy. Our findings suggested that PKMYT1 is a promising target to improve the radiosensitivity of lung adenocarcinoma.

## Data Availability Statement

The datasets generated for this study can be found in the data is available at NCBI GEO under accession numbers GSE33532, GSE32863, and GSE43458.

## Ethics Statememt

This study was approved by the Ethics Committee of The First Hospital of China Medical University.

## Author Contributions

HL analyzed the data and drafted the manuscript. JL, YY, QQ, and GL provided suggestions and approved the final manuscript.

## Conflict of Interest

The authors declare that the research was conducted in the absence of any commercial or financial relationships that could be construed as a potential conflict of interest.

## Abbreviations

DAVID: the Database for Annotation Visualization and Integrated Discovery
GO: Gene Ontology
GEPIA: the Gene Expression Profiling Interactive Analysis
KEGG: Kyoto Encyclopedia of Genes and Genomes
LUAD: lung adenocarcinoma
PPI: protein-protein interaction
OS: overall survival.
